# *In silico* Analysis Revealed High-risk Single Nucleotide Polymorphisms in Human Pentraxin-3 Gene and their Impact on Innate Immune Response against Microbial Pathogens

**DOI:** 10.3389/fmicb.2016.00192

**Published:** 2016-02-23

**Authors:** Raman Thakur, Jata Shankar

**Affiliations:** Department of Biotechnology and Bioinformatics, Jaypee University of Information TechnologySolan, India

**Keywords:** PTX-3, nsSNPs, *In silico*, *Aspergillus fumigatus*, galactomannan, lipopolysaccharide

## Abstract

Pentraxin-3 (PTX-3) protein is an evolutionary conserved protein that acts as a soluble pattern-recognition receptor for pathogens and plays important role in innate immune response. It recognizes various pathogens by interacting with extracellular moieties such as glactomannan of conidia (*Aspergillus fumigatus*), lipopolysaccharide of *Pseudomonas aeruginosa, Streptococcus pneumonia* and *Salmonella typhimurium*. Thus, PTX-3 protein helps to clear these pathogens by activating downstream innate immune process. In this study, computational methods were used to analyze various non-synonymous single nucleotide polymorphisms (nsSNPs) in PTX-3 gene. Three different databases were used to retrieve SNP data sets followed by seven different *in silico* algorithms to screen nsSNPs in PTX-3 gene. Sequence homology based approach was used to identify nsSNPs. Conservation profile of PTX-3 protein amino acid residues were predicted by ConSurf web server. In total, 10 high-risk nsSNPs were identified in pentraxin-domain of PTX-3 gene. Out of these 10 high-risk nsSNPs, 4 were present in the conserved structural and functional residues of the pentraxin-domain, hence, selected for structural analyses. The results showed alteration in the putative structure of pentraxin-domain. Prediction of protein–protein interactions analysis showed association of PTX-3 protein with C1q component of complement pathway. Different functional and structural residues along with various putative phosphorylation sites and evolutionary relationship were also predicted for PTX-3 protein. This is the first extensive computational analyses of pentraxin protein family with nsSNPs and will serve as a valuable resource for future population based studies.

## Introduction

Single nucleotide polymorphisms (SNPs) are the single base change in coding or non-coding DNA sequence and are present in every 200–300 bp in human genome (Lee et al., 2005). So far, 5,000,000 SNPs have been identified in the coding region of human population responsible for genetic variation (Rajasekaran et al., 2008). Among all SNPs, non-synonymous SNPs (nsSNPs) are present in exonic part of genome, which often leads to change in amino acid residues of gene product. Substitution of amino acid in protein may alter the stability and protein structure, affecting the function. Alteration in protein function due to nsSNPs has been reported to various diseases in human (George Priya Doss et al., 2008; Dabhi and Mistry, 2014; Nagasundaram et al., 2015). Recently many studies have been focused on deleterious nsSNPs associated with immunity related genes especially genes associated with innate immune response (Davis et al., 2010; Lin et al., 2012; Ovsyannikova et al., 2014). In addition, recently limited studies have been carried using *in silico* approaches to understand how nsSNP could be deleterious at protein/functional level (Dabhi and Mistry, 2014; Kelly and Barr, 2014).

Human pentraxin proteins are conserved pattern recognition protein receptors, that play a crucial role in innate immunity response (Mantovani et al., 2008). Based on the size, pentraxin proteins are divided into long pentraxins (PTX-3, PTX-4, Neuronal pentraxin-1 and 2) and short pentraxins (C-reactive protein and serum amyloid protein; Garlanda et al., 2002). Pentraxin proteins contain conserved pentraxin-domain at C-terminus and multiple domain sites at N-terminus. C-reactive protein and serum amyloid are well studied member of pentraxin protein family and they have the ability to recognize polysaccharides from bacteria such as *Streptococcus pneumonia* and provide resistance against microbial infections (Garlanda et al., 2002; Martinez de la Torre et al., 2010). Furthermore, PTX-3 protein is a key soluble pattern recognition receptor of innate immunity in lung infections caused by *Salmonella typhimurium, Pseudomonas aeruginosa* and *Aspergillus fumigatus* (Garlanda et al., 2002; Chiarini et al., 2010; Thakur et al., 2015). Invasive infection has been associated to *Aspergillus* species at different organ sites (Kidney, Brain) with predominantly T_H_1 immune response (Anand et al., 2013, 2015; Thakur et al., 2015).

PTX-3 protein is produced by various immune cells particularly dendritic cells, macrophages and neutrophils as 10–20 subunit multimer protein in response to various inflammatory mediators such as bacterial lipopolysaccharide, galactomannan of *A. fumigatus* conidia, interlukin-1, and tumor-necrosis factor-α, respectively (Rovere et al., 2000; Balhara et al., 2013). N-terminus and C-terminus of PTX-3 protein overlap and act as pathogen recognition receptors. They recognize various moieties of microbes and facilitate their opsonization (Garlanda et al., 2002). Pentraxin conserved domain binds with C1q and activates the classical complement pathway to signal innate immunity in response to infection (Inforzato et al., 2006). PTX-3 protein inhibits the colonization of various microorganisms in the respiratory tract and lung that includes fungi such as *Candida albicans* and *A. fumigatus* and virus like *influenza virus* (Reading et al., 2008; Cunha et al., 2014). It has been previously reported as a vital component of antifungal innate immune response (Salvatori and Campo, 2012). PTX-3 protein is also suggested as a biomarker to monitor immunopathological conditions of the patients (Balhara et al., 2013). Despite, the importance of PTX-3 gene and its high polymorphic nature it is still unclear how nsSNPs could affect its protective functions against human pathogens, which colonize in respiratory tract and lungs. Taking these into consideration, multiple *in silico* methods were chosen to identify nsSNPs in pentraxin-3 gene, and to predict structure, and function of pentraxin protein. Our analysis showed 10 high- risk nsSNPs in pentraxin-domain. Out of them, 4 nsSNPs were present in highly conserved amino acid residues and such pentraxin-domain variants are considered for putative structure analysis that resulted in alteration in domain possibly affecting their functions. The *in silico* analysis of pentaxin-3 gene of innate immunity paved a foundation for population based studies on predicted deleterious nsSNPs.

## Materials and methods

### SNP data mining for pentaxin-3 human gene

SNPs and protein sequence for PTX-3 gene were collected from different web-based data sources such as dbSNP databases: the NCBI dbSNP database (https://www.ncbi.nlm.nih.gov/SNP), UniProt data base (UniProtKB ID P26022) and the Ensembl genome browser (http://www.ensembl.org/index.html; Sherry et al., 2001; Flicek et al., 2014; Uniport, 2014). These SNPs were used for bioinformatics analysis.

### Prediction of functional consequences of nsSNPs

nsSNPs were collected from retrieved SNPs and their functional consequences were predicted by using different *in silico* algorithms: Sorting Intolerant from Tolerant (SIFT) server was used to predict the deleterious effects of nsSNPs (http://sift.jcvi.org/). SIFT utilizes the sequence homology of the proteins and by aligning the natural occurring nsSNPs with paralogous and orthologous protein sequences to predict the deleterious effect of nsSNPs. The SIFT score less than 0.05 indicates the harmful effect of nsSNPs on protein function. Another algorithm used for the prediction of functional consequences of nsSNPs was polyphen-2 (http://genetics.bwh.harvard.edu/pph2). Polyphen-2 software uses the protein sequence as well as the amino acid variant position in protein sequence to predict the effect of nsSNP on protein structure and function.

If there is a change in amino acid or mutation in protein sequence, it is evaluated as “possibly damaging” (probabilistic score > 0.15), “probably damaging” (probabilistic score > 0.85) and “benign” (remaining mutations). PolyPhen-2 also calculates the PISC score for each amino acid substitution in protein. The PSIC score difference among variants directly indicates the functional consequences of nsSNPs on protein function. Another algorithms used for the prediction of nsSNPs effects on protein function were SNAP (https://www.broadinstitute.org/mpg/snap/), PhD-SNP (http://snps.biofold.org/phd-snp/phd-snp.html), MAPP (http://www.ngrl.org.uk/Manchester/page/missense-prediction-tools) and PANTHER (http://www.pantherdb.org/). In total, seven different SNP prediction algorithms were used. nsSNPs predicted to be deleterious by at least five different *in silico* SNP algorithms were categorized as high-risk nsSNPs. Because each algorithm uses different parameters to assess the nsSNPs, hence, nsSNPs with more positive results in SNP algorithms are more likely to be deleterious. 10 nsSNPs predicted to be deleterious by SNP prediction algorithms were subjected for further analyses.

### Conservation profile and phylogenetic analysis of PTX-3 protein

To carry out phylogenetic analyses, human protein sequence for PTX-3 (GenBank Accession Number: NP_002843.2) and protein sequences for other eight species of Hominidae family such as *Pan troglodytes* (XP_516838.2), *Mesocricetus auratus* (XP_005077995.1), *Gorilla gorilla gorilla* (XP_004037950.1), *Cavia porcellus* (XP_003476359.1), *Bos Taurus* (NP_001069727.1), *Bison bison bison* (XP_010838164.1), and for Muridae family, *Mus musculus* (CAA58580.1) and *Rattus norvegicus* (NP_001103006.1) were retrieved from the National Center for Biotechnology Information (NCBI) and subjected for their evolutionary conservation. Protein sequence alignment was performed using ClustalX software version 1.8331 (http://www.clustal.org/) and by using multiple sequence comparison by log-expectation (MUSCLE; Edgar, 2004; Larkin et al., 2007). Multiple alignment files saved by ClustalX and MUSCLE in the ClustalX and MUSCLE format were converted to the MEGA format (^*^.meg) using the MEGA version 6 (http://www.megasoftware.net/; Tamura et al., 2013). Evolutionary conservation of amino acid residues in PTX-3 protein was carried out by ConSurf web server (consurf.tau.ac.il/; Ashkenazy et al., 2010). Phylogenetic analysis was performed by the Maximum Parsimony (MP) method using MEGA version 6 that helps to construe ancestral affiliations and calculate the rates of molecular evolution. The corresponding parameters of the MP algorithm were set at “complete deletion,” and the “protein: p-distance” model. Bootstrap method (1000 bootstrap replicates to generate statistically significant phylogenetic tree)' was used (Tamura et al., 2013).

### Prediction of change in protein stability due to nsSNPs

To predict the change in protein stability due to mutation, we used I-Mutant version 2, a support vector machine based tool server. I-Mutant version 2 predict the Gibbs free energy change (DDG) by subtracting the mutated protein unfold Gibbs free energy (ΔG) from wild type proteins unfold Gibbs free energy (ΔG). Prediction of energy change can be performed by use of either protein sequence or structure.

I-Mutant version 2 also predict the sign of decrease (Dec.) or increase (Inc.) in Gibbs free energy with Reliability Index (RI) for change in amino acid, where RI-0 indicates lowest reliability and RI-10 indicates highest reliability. The value of change in free energy (DDG) below 0 (< 0) indicates decrease in protein stability and value higher than 0 (>0) indicates increase in protein stability. During prediction of energy change, the pH and temperature was set as 7 and 25°C, respectively, for all nsSNP submissions.

### Prediction of nsSNPs positions in different protein domains

To locate the nsSNPs position in different domains of PTX-3 protein structure, we used NCBI Conserved Domain Search tool (http://www.ncbi.nlm.nih.gov/Structure/cdd/wrpsb.cgi) and InterPro (http://www.ebi.ac.uk/interpro/; Hunter et al., 2009; Marchler-Bauer et al., 2015). These tools take either protein sequence in FASTA format or protein ID as query sequence to deduce their domain and motifs. Both of these tools provide functional analysis of different proteins by classifying them into different families as well as domains or important sites.

### Molecular effects of high-risk nsSNPs on protein structure

Three dimensional (3D) structure analyses were done for pentraxin-domain (wild type) and each of the high-risk nsSNP located within this domain. Homology modeling of pentraxin-domain was carried out to predict 3D-structure. The protein sequence of human PTX-3 protein was retrieved from UniProt data base (UniProtKB ID P26022). Homology modeling was done by Web based server Phyre2 (http://www.sbg.bio.ic.ac.uk/phyre2). This server uses Hidden Markov Method to generate alignments of submitted protein sequence against proteins with published protein structure. The resulting alignments are then used to produce homology based models of the query sequence to predict its three-dimensional structure. This server also uses an ab-initio folding simulation called Poing to model region of a query sequence with no detectable similarities to known structures. It combines multiple templates of known structure to produce final model of the query sequence. The 3D-structure model for pentraxin-domain was developed by submitting FASTA sequence of pentraxin-domain region to Phyre2 server under intensive mode. The Phyre2 server has used these templates having PDB ID (% identity); {1sac (24%), 1b09 (24%), 4pbo (23%), and 3flp (22%)} for the modeling of 3D-structure of pentraxin-domain. When the degrees of similarity of these templates were analyzed using BLASTp, human's templates 1sac and 4pbo showed 51% similarity with 70% positives. The structure refinement of the predicted model was carried out by ModRefiner by submitting PDB file of model structure (Xu and Zhang, 2011). Then energy minimization of model was performed with YASARA force field minimization tool to improve quality of predicted model of pentraxin-domain (Krieger et al., 2009). Further, model structure of pentraxin-domain was validated by using RAMPAGE server (http://mordred.bioc.cam.ac.uk/~rapper/rampage.php). RAMPAGE is a popular program used to check the stereo-chemical quality of a protein structure. A Phi/Psi Ramachandran plot was obtained from RAMPAGE to validate the structure of pentraxin-domain.

### Modeling of amino acid substitution and energy minimization

To generate the mutated models of pentraxin-domain for corresponding amino acid substitutions, Swiss-PDB Viewer was used (Guex and Peitsch, 1997). The PDB model generated from Phyre2 server was used for mutated model generation of pentraxin domain. Swiss-PDB “mutation tool” was used to replaces the wild type amino acid with a new amino acid.” The mutation tool facilitates the replacement of the native amino acid by the “best” rotamer of the new amino acid. The “.pdb” files were saved for all the models. Then to improve the quality of predicted model, energy minimization was performed with the YASARA force field minimization server (Krieger et al., 2009). The 3D-structure models were viewed using PyMOL (https://www.pymol.org/). Further, Tm-score, and Root Mean Square Deviation (RMSD) was estimated for each mutated models using TM-Align (http://zhanglab.ccmb.med.umich.edu/TM-align/; Zhang and Skolnick, 2005).

### Prediction of putative post-translation modification sites in PTX-3 protein

Different post-translation modification sites like wise putative ubiquitylation, sumoylation, phosphorylation, and glycosylation sites were predicted using different programs. Putative phosphorylation sites were predicted using NetPhos 2.0 (http://www.cbs.dtu.dk/services/NetPhos/) and GPS 2.1 (http://gps.biocuckoo.org/). For GPS 2.1 analysis, cut-off values ranging from 0.776-11 were selected. In NetPhos 2.0, threonine, serine, and tyrosine residues with a score of 0.5 were considered phosphorylated (Blom et al., 1999; Xue et al., 2011). Putative sumoylation sites were predicted using SUMOsp 2.0 (http://sumosp.biocuckoo.org/) program and SUMOplot (http://www.abgent.com/sumoplot) program. For SUMOplot, only high probability motifs with a score 0.5 were considered sumoylated. Medium level threshold with a 2.64 cut-off value was selected for SUMOsp 2.0 analysis, respectively (Gill, 2003; Xue et al., 2006). Whereas, putative ubiquitylation sites were predicted by using BDM-PUB (bdmpub.biocuckoo.org) and UbPred (www.ubpred.org) programs (Radivojac et al., 2010). For BDM-PUB, the only balanced cut-off value option was taken and in case of UbPred, the lysine amino acid having 0.62 score was considered as ubiquitylated in PTX-3 protein.

### Prediction of protein–protein interactions for PTX-3 protein

Protein-protein interaction networks are important to reveal and interpret all functional interaction among cellular proteins. In the current study, the online database resource “STRING” Search Tool for the Retrieval of Interacting proteins (http://string-db.org/) was used. This tool provides unique coverage and ease of access to both theoretical and experimental interaction evidence of PTX-3 protein of human. The input options for STRING database include protein sequence, protein name, and multiple sequences. STRING database is presently equipped with 5,214,234 proteins belonging to 1133 organisms. The interaction studies were performed in various modes. The different modes include evidence view, confidence view, interactive mode, and action view to deduce the most appropriate interactions among nodes in PTX-3 protein interaction network (Szklarczyk et al., 2011).

## Result and discussion

### Retrieval of SNP datasets

SNPs from dbSNP (National Center for Biotechnology Information, UniProt database, and Ensembl genome browser; Sherry et al., 2001; Flicek et al., 2014; Uniport, 2014) showed human PTX-3 gene contains 32 SNPs in 3′UTR region, 4 SNPs in 5′UTR region, 170 SNPs in non-coding region (Intron), and 65 missense variants. We subjected these 65 missense mutations or nsSNPs to various *in silico* SNP prediction algorithms that are summarized in Table 1, which characterizes nsSNPs as neutral or deleterious to structure and function of PTX-3 protein.

**Table 1:** **Predicted results for nsSNPs in PTX-3 gene using different algorithms**.

**Prediction**	**nsSNPs (%)**						
	**SIFT**	**PP-2**	**PP-1**	**MAPP**	**PhD-SNP**	**SNAP**	**PANTHER**
Deleterious	25(38)	27(41)	21(32)	37(57)	18(28)	29(45)	7(11)
Neutral	40(62)	38(59)	44(68)	28(43)	47(72)	36(55)	58(89)

*It shows percentage of deleterious and neutral nsSNPs out of total nsSNPs (65) by seven different algorithms; PP-2, polyphen-2, and PP-1, polyphen-1*.

### Analysis of functional consequences of nsSNPs

Seven *in silico* SNPs prediction algorithms were used; SIFT, PP-2, PP-1, MAPP, PhD-SNP, SNAP and PANTHER (see Supplementary Table 1). SIFT analysis uses sequence homology and characterized the effect of amino acid substitution on protein function (Capriotti et al., 2006; Bromberg et al., 2008; Kumar et al., 2009; Adzhubei et al., 2013). As each of these algorithms use different parameters to assess the nsSNPs to be deleterious or neutral, the percentage of deleterious or neutral nsSNPs in PTX-3 by different algorithms have been summarized in Table 1.

**Figure 1:**
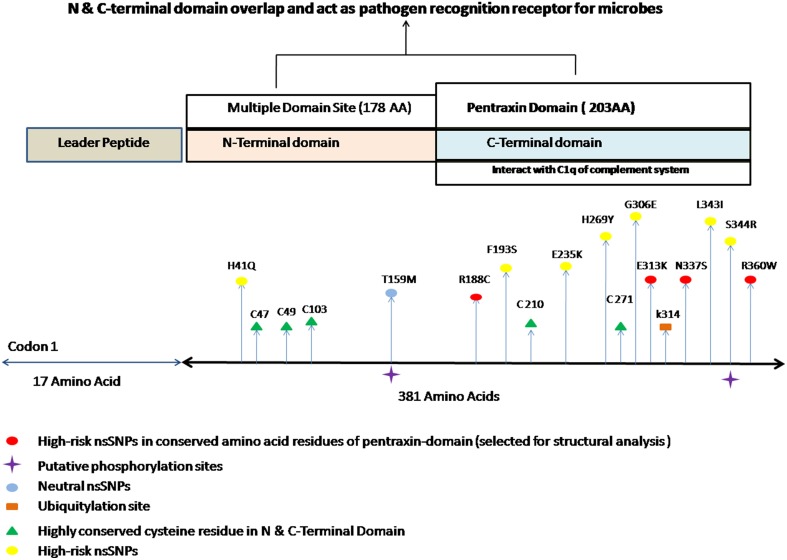
**Different putative functional sites in PTX-3 protein**. It indicates the approximate location of different high risk nsSNPs along with different post-translation modification sites. It also shows the highly conserved cysteine amino acid residue in N and C-terminal domain of PTX-3 protein. PTX-3 contain N-terminal site having 178 amino acid and C-terminal site having 203 amino acid residues, respectively. N-terminus contains multiple domain sites and C-terminus domain contains pentraxin-domain. Pentraxin-domain interacts with C1q component of complement system to activate innate immune response (Balhara et al., 2013). N- and C-terminal domain overlap each other, interacts with various ligands and also act as PRR (pathogen recognition receptors), which recognizes *A. fumigatus* conidial glactomannan (Salvatori and Campo, 2012).

Furthermore, by using NCBI Conserved Domain Search (http://www.ncbi.nlm.nih.gov/Structure/cdd/wrpsb.cgi; Marchler-Bauer et al., 2015) and InterPro (http://www.ebi.ac.uk/interpro/) (Hunter et al., 2009) tools, pentraxin-domain was predicted in PTX-3. We found 10 deleterious nsSNPs present in pentraxin-domain predicted by at least five different *in silico* SNP prediction algorithms (Table 2 and Figure 1) and these were considered as high-risk nsSNPs. It has been reported, that N-terminal domain overlaps with C-terminal domain (pentraxin-domain) and both of them act as PRR (Pathogen Recognition Region; Martinez de la Torre et al., 2010). nsSNPs E313K, R188C, N337S, and R360W present in pentraxin-domain are highly conserved functional residues (Table 3) and hence, selected for structure prediction followed by comparison analysis with the wild type structure.

**Table 2:** **Prediction of deleterious nsSNP in PTX-3 gene**.

**nsSNP ID**	**Mutation position**	**Domain**	**Deleterious prediction**
rs564774580	R188C	PTX	5
rs529759691	F193S	PTX	5
rs532972316	E235K	PTX	5
rs190837481	H269Y	PTX	5
rs144979346	G306E	PTX	5
rs4478039	E313K	PTX	5
rs76994524	N337S	PTX	5
rs146705881	L343I	PTX	5
rs140073706	S344R	PTX	5
rs138818541	R360W	PTX	5

*It represents the use of seven different SNP algorithms with different parameters to evaluate the nsSNPs. nsSNPs predicted to be deleterious by at least five different in silico SNP algorithms were categorized as high-risk nsSNPs. Because each algorithm uses different parameters to assess the nsSNPs, hence, nsSNPs with more positive results in SNP algorithms are more likely to be deleterious. 10 nsSNPs predicted to be deleterious in pentraxin-domain by at least five different SNP algorithms (Figure 1)*.

**Table 3:** **Conserved amino acids in PTX-3 protein that coincide in location with high-risk nsSNPs**.

**nsSNPs ID**	**Amino acid position**	**CS**	**ConSurf prediction**
rs564774580	R188C^*^	9	Highly conserved and Exposed (F)
rs529759691	F193S^*^	8	Highly conserved and Buried
rs532972316	E235K^*^	9	Highly conserved and Exposed (F)
rs190837481	H269Y^*^	9	Buried (S)
rs144979346	G306E^*^	9	Highly conserved and Buried (S)
rs4478039	E313K^*^	9	Highly conserved and Exposed (F)
rs76994524	N337S^*^	9	Highly conserved and Buried (S)
rs146705881	L343I^*^	9	Highly conserved and Buried (S)
rs140073706	S344R^*^	7	Exposed
rs138818541	R360W^*^	9	Highly conserved and Exposed (F)

*It indicates the conservation score of different amino acid residues calculated by ConSurf: Conservation score (1–4 variable, 5-Average, 6–9 conserved); F, functional; S, structural predicted sites by use of ConSurf Web Server. Asterisk indicates mutated sites at pentraxin conserved domain*.

Recent studies showed nsSNP A48D (rs3816527) in PTX-3 gene is associated with colonization of *Pseudomonas aeruginosa* in cystic fibrosis patients (Chiarini et al., 2010). Cunha et al., has reported nsSNP A48D (rs3816527) in PTX-3 gene in patients of invasive aspergillosis that undergo hematopoietic stem cell transplantation and suggested substitution of amino acid may affect interaction of PTX-3 protein with other proteins due to change in electrostatic potential (Cunha et al., 2014). In our analyses, we also observed nsSNP A48D (rs3816527) in PTX-3 as a deleterious nsSNP by only SNAP nsSNP prediction algorithm.

### Conservation profile of high-risk nsSNPs and phylogenetic analysis of PTX-3 protein

Conserved amino acids in proteins are involved in various cellular processes in a biological system including genome stability (Greene et al., 2001). Amino acids that are located at enzymatic sites or required for protein-protein interaction become more conserved than other amino acids in protein (Williamson et al., 2013). Due to this reason, the nsSNPs that are located in conserved region are more deleterious as compared to nsSNPs that are located in variable regions in a protein because they destabilize their structure and function. For evaluating the harmful effects of 10 high-risk nsSNPs in the PTX-3 protein, we predicted the evolutionary conservation profile of PTX-3 gene using ConSurf web browser, which use Bayesian method to determine evolutionary conserved amino acid residues in proteins and also identify functional and structural residues (Ashkenazy et al., 2010). ConSurf analysis predicted 10 highly conserved amino acid residues in pentraxin-domain of PTX-3 protein (Table 3, Supplementary Figure 1) and two amino acid residues in N-terminal domain site (multidomain site; Table 3). We also observed 4 highly conserved amino acids (R188C, E235K, E313K, and R360W) that are exposed functional-residues and 3 highly conserved amino acids (G306E, N337S, and L343I) that are buried structural-residues in PTX-3 protein. Furthermore, to identify the functional and structural sites in protein sequence ConSurf software was used, which uses evolutionary conservation data along with solvent accessibility prediction. Using these parameters, highly conserved amino acids (conservation score 8–9) were predicted to be functional and/or structural amino acid residues in PTX-3 protein based on their location relative to core-protein or protein-surface. Based on analyses, our data suggest that nsSNPs E313K, R188C, N337S, and R360W (Tables 2, 4) may alter the structure and function of PTX-3 protein especially to the pentraxin-domain (C-terminal domain). Among four high-risk nsSNP, we found R188C present in uterine corpus endometrioid carcinoma patients, amino acid mutation id-COSM1040427 available at catalog of somatic mutation in cancer (COSMIC) genome browser.

**Table 4:** **High-risk nsSNPs taken for structure analysis**.

**nsSNP ID**	**Mutation**	**Deleterious prediction**	**ConSurf prediction**
rs4478039	E313K	6	9 Exposed (F)
rs1536891	R188C	6	9 Exposed (F)
rs76994524	N337S	5	9 Buried (S)
rs138818541	R360W	6	9 Exposed (F)

*This table indicated the high-risk nsSNPs that are coincide with in pentraxin-domain of PTX-3 protein and highly deleterious predicted by more than four algorithms. Conservation score was calculated using ConSurf. Conservation (1–4 variable, 5-Avg, 6–9 conserved)*.

**Figure 2:**
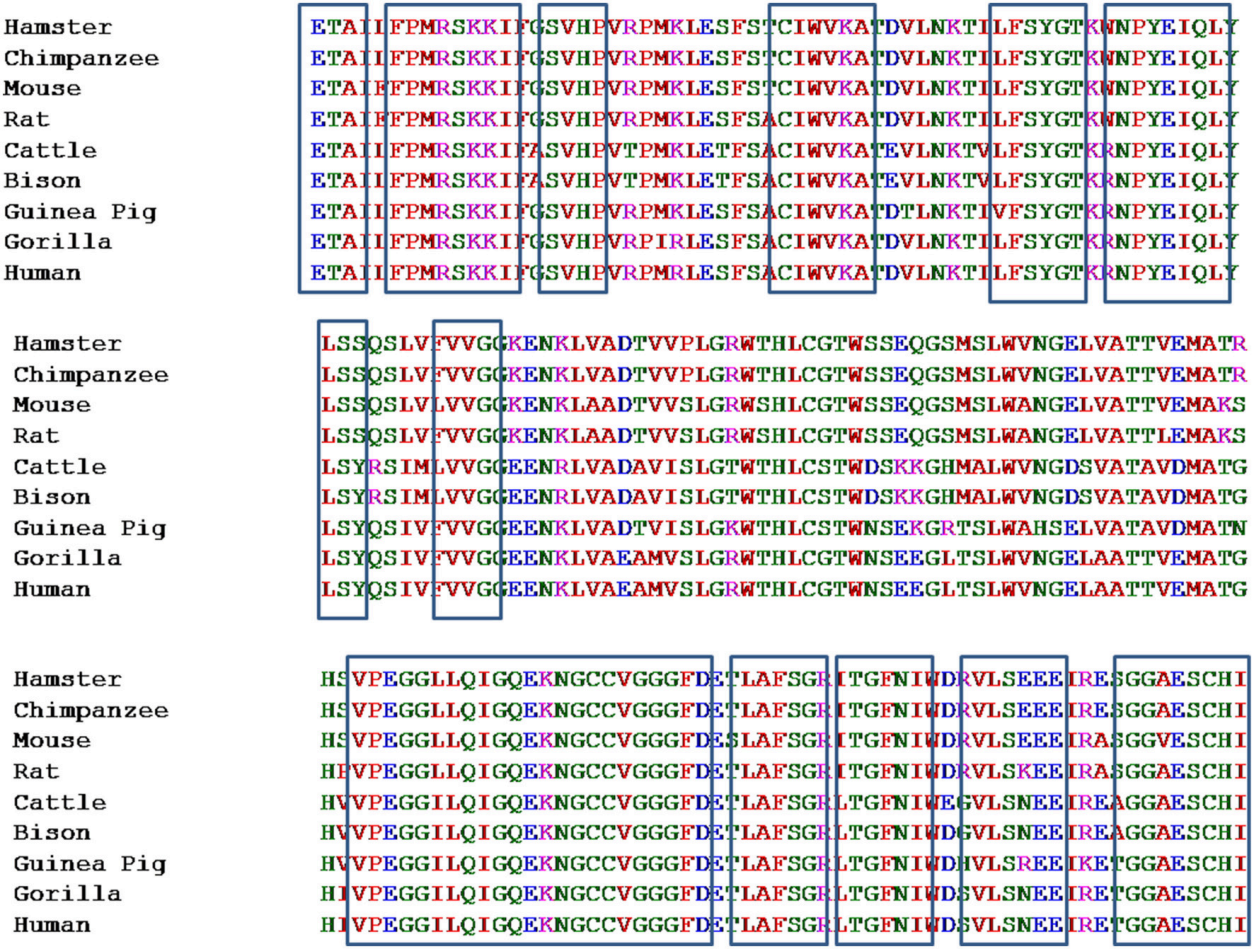
**Multiple sequence alignment of pentraxin-domain of PTX-3 protein among nine species taking human PTX-3 protein as reference**. It represents the Multiple Sequence Alignment of conserved pentraxin-domain of PTX-3 protein of 9 different species. This was carried out using multiple sequence comparison by log-expectation (MUSCLE), which uses log-expectation scores and fast fourier transform methods, respectively. Program was used with default parameters. Human PTX-3 sequence was taken as a reference and is shown at the bottom of MSA. Areas in boxes represent various conserved regions in pentraxin domain.

Pentraxin-3 gene is present on chromosome number 3 (q22–28) and contains three exons. First two exons encode for leader and N-terminus, whereas third exon encodes for C-terminus known as pentraxin-domain. Human pentraxin-domain of PTX-3 protein is highly conserved as compared to N-terminal domain (Breviario et al., 1992; Martinez de la Torre et al., 2010). The human and mouse PTX-3 proteins share 93% sequence similarity (Garlanda et al., 2002). Evolutionary relationship of PTX-3 proteins among nine different primates' species was carried out using human PTX-3 protein as a reference sequence. The multiple sequence alignment (MSA) generated form ClustalX and MUSCLE tools for the PTX-3 protein sequences were quite similar and various conserved regions were identified among nine species including human (Figure 2). In Figure 2 the highly conserved regions are shown in black box. Also, multiple sequence alignment showed PTX-3 protein is conserved throughout nine species (Figure 2).

**Figure 3:**
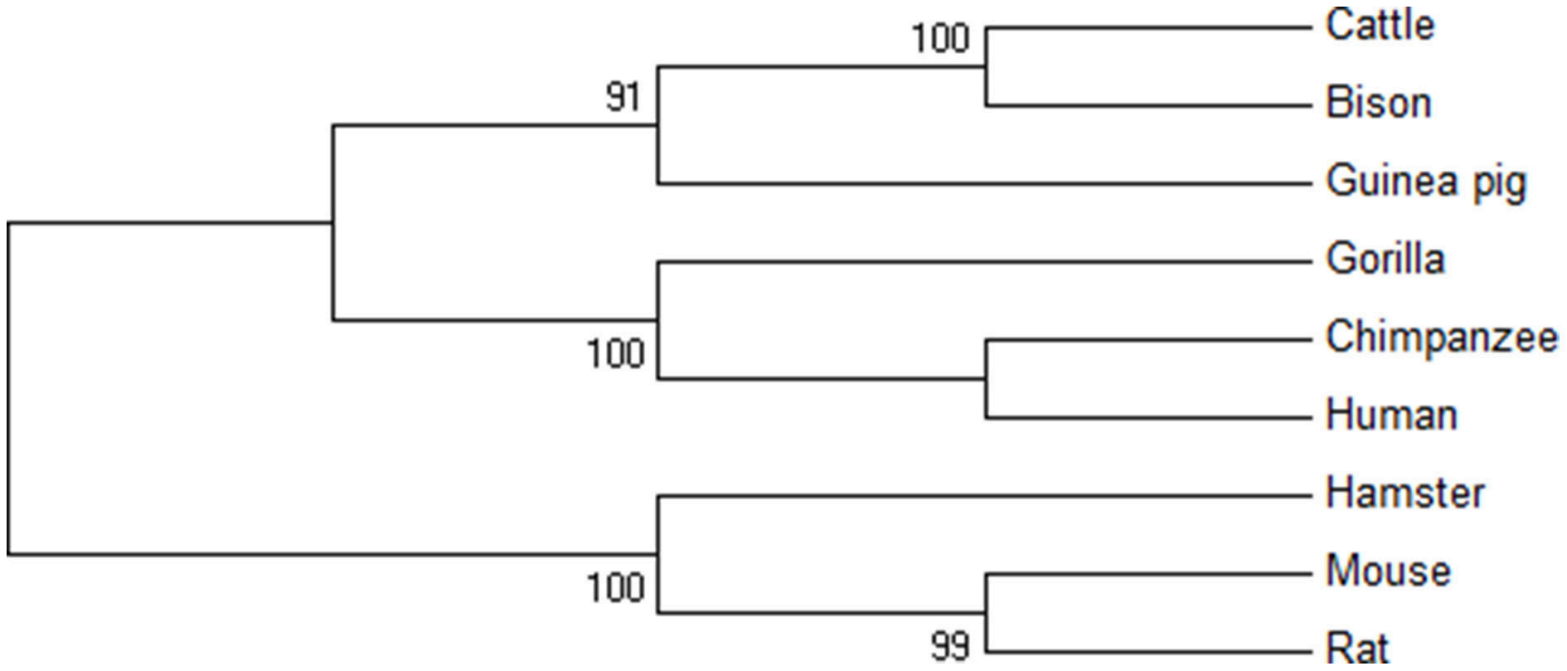
**Phylogenetic analysis of PTX-3 protein**. A phylogenetic tree of the amino acid sequences of PTX-3 protein was constructed using the maximum parsimony (MP) method. The value on each branch is the estimated confidence limit (expressed as a percentage) for the position of the branches, as determined by bootstrap analysis. Only values exceeding 70% are shown.

Furthermore, phylogenetic analysis was done for PTX-3 protein between nine species of primates including human (Figure 3). The phylogenetic tree generated form MEGA version 6 help to understand the evolutionary relationship among different species. Evolutionary tree demonstrates that human and chimpanzees lies close to each other suggesting that the PTX-3 gene is identical in these primates and could be originated from same ancestors. From phylogenetic tree analysis; we concluded that the PTX-3 is highly conserved in primates including humans (Figure 3).

### Comparative structure modeling of deleterious high-risk nsSNPs

**Figure 4:**
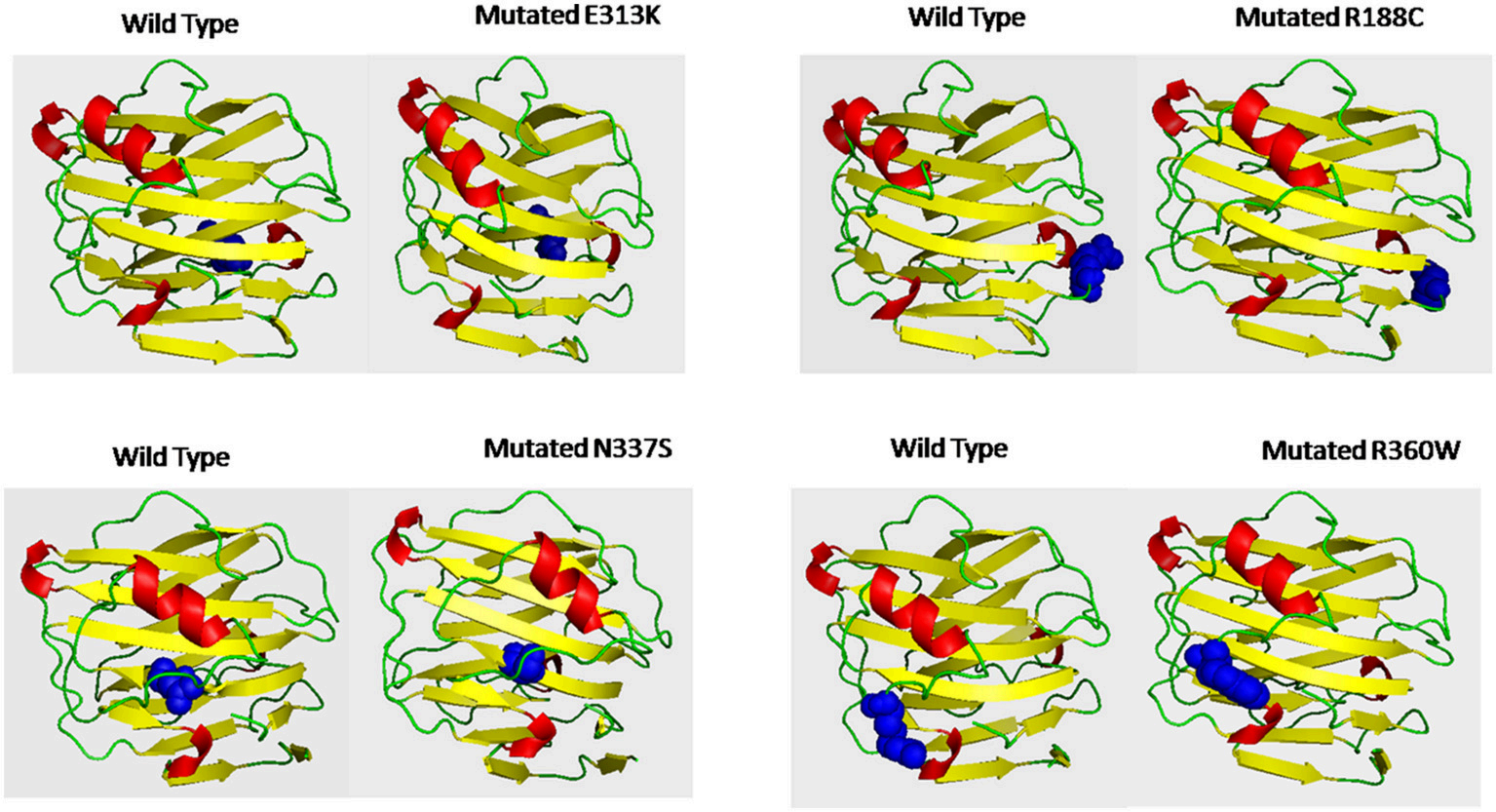
**Shows the 3D-putative structure of wild type pentraxin-domain and mutated type domain generated by Phyre-2 version 2.0, which search multiple sequence databases and build 3D-structure based on homolog of known structure**. Putative models were viewed by PYMOL and SWISSPDB viewer. Putative 3-D structures were drawn for four high-risk nsSNPs located in pentraxin-domain. Left side of each box shows 3-D structure of wild type pentraxin-domain and at right side shows 3-D putative structure of mutated pentraxin-domain. The location of each mutated amino acid in mutated putative 3-D structures and in wild type pentraxin-domain shown in blue color.

Deleterious high risk nsSNPs predicted by analysis were subjected to 3D-structure analysis. The ability of proteins to perform various functions or to interact with other molecules depends upon its tertiary structure (Alshatwi et al., 2011). To predict the 3D-structure of pentraxin-domain homology modeling was performed by using Phyre-2 server. Following templates e.g., 4pbo, 1b09, 1sac, and 3flp has been used by Phyre-2 server for the modeling of 3D-structure of pentraxin-domain. 4pbo is a zebrafish short-chain pentraxin proteins, 1b09 is a human C-reactive protein, 1sac is a human serum amyloid p-component, and 3flp is a heptameric SAP like pentraxin protein from *Limulus polyphemus*. These proteins are short-chain pentraxin proteins, where as PTX-3 is a long-chain pentraxin due to additional N-terminal domain. C-terminal domain (Pentraxin-domain) of PTX-3 is homologous to short-chain pentraxin proteins and are involved in recognition of pathogenic bacteria and fungi to activate the classical complement pathway via C1q, a complement pathway component (Inforzato et al., 2013; Chen et al., 2015). Predicted model structure of pentraxin-domain was submitted to structure refinement and energy minimization using ModRefiner and YASARA force field minimization server (Krieger et al., 2009; Xu and Zhang, 2011). The energy minimization repaired the distorted geometries of pentraxin-domain. After this step, model validation was done using RAMPAGE. The Ramachandran plot was obtained from RAMPAGE for energy minimized pentraxin-domain structure predicted by Phyre-2. The structure stability of pentraxin-domain was confirmed by Ramachandran plot. 197 (98%) residues were found in favored region, 3 (1.5%) residues were in allowed region, and 1 (0.5%) residue was found in outlier region. Further, to generate mutated model structure of pentraxin-domain for corresponding amino acid substitutions, Swiss PDB viewer was used (Figure 4). Mutated model structure of high-risk nsSNPs (R188C, E313K, N337S, and R360W) was subjected to RAMPAGE for stability assessment (Supplementary Figure 2 showed RAMPAGE for wild type PTX-3 model protein structure and Supplementary Figures 3–6 showed RAMPAGE for mutated model structure of PTX-3 protein having R188C, E313K, N337S and R360W, respectively). Results of the Ramachandran plot analysis for each of the high-risk nsSNP model structure was given in Table 5. To extend these analyses, we further calculated the root mean square deviation (RMSD) and Tm-score for R188C, E313K, N337S, and R360W high-risk nsSNPs. RMSD is used to measured average distance between alpha carbon backbone of wild type and mutant models of protein, where Tm-score is used to determine topological similarity between wild type and mutant model (Zhang and Skolnick, 2005; Table 6). A higher RMSD value indicated the deviation in mutant structure as compared to wild type. The maximum RMSD value was found in R360W (0.99) followed by R188C (0.97) and N337S (0.95). This result further indicates that the high-risk nsSNPs significantly alter the structure stability of pentraxin-domain of PTX-3 protein.

**Table 5:** **Analysis of Ramachandran plot of modeled structures by using RAMPAGE server**.

**Model**	**Residues in most favored regions**		**Residues in allowed regions**		**Residue in outlier region**	
	**No. of residues**	**% of residues**	**No. of residues**	**% of residues**	**No. of residues**	**% of residues**
Wild type	197	98	3	1.5	1	0.5
R188C	189	94.01	10	5	2	1
E313K	190	94.5	8	4	3	1.5
N337S	188	93.5	10	5	3	1.5
R360W	186	92.5	12	6	3	1.5

**Table 6:** **TM-score and RMSD (Å) value for the high risk nsSNPs in pentraxin-domain of PTX-3 protein calculated by use of TM-align calculator**.

**nsSNP ID**	**Mutation position**	**TM-Score**	**RMSD(Å)**
rs1536891	R188C	0.97	0.97
rs4478039	E313K	0.98	0.92
rs76994524	N337S	0.98	0.95
rs138818541	R360W	0.97	0.99

*It represents, that Tm-score is used to assess topological similarity between wild type and mutant protein model. TM-align first generate optimized residue-to-residue alignment based on structural similarity using dynamic programming iterations. An optimal superposition of the two structures, as well as the Tm-score value which scales the structural similarity, will be returned. TM-score has the value in (0, 1), where 1 indicates a perfect match between two structures. Whereas, RMSD (root mean square deviation) is used to measure distance between α-carbon backbone of wild type and mutant models. A higher RMSD value indicates greater deviation between wild type and mutant structure*.

Pentraxin-domain of PTX-3 protein is essential for its function as it interacts with various components of innate immunity, strongly with C1qA, C1qC, and CFH to activate the classical complement pathway of innate immunity (Balhara et al., 2013). If the structure of pentraxin-domain is altered, could be possible that it may not be able to interact with these components of complement pathway. Thus, humans with these nsSNPs in their genome may be susceptible to various pathogens particularly *A. fumigatus, P. aruogenisa, S. pneumonia, S. typhimurium*, and *Influenza virus* (Reading et al., 2008; Chiarini et al., 2010; Cunha et al., 2014). It is also possible that these high-risk nsSNPs may impair the function of PTX-3 protein as pathogen recognition receptor recognizes several moieties present on cell wall of the pathogens and allow these pathogens for clearance by phagocytic cells.

Further, we used I-mutant to identify the stability of PTX-3 protein containing high-risk nsSNPs. I-mutant is a machine based tool to measure the free energy change caused by single amino acid in protein sequence (Capriotti et al., 2005). High-risk nsSNPs N337S and R360W were predicted to be less stable than the wild type protein, with free energy change values of −0.81 and −0.34, respectively, and having high conserved value predicted by ConSurf (Table 7).

**Table 7:** **Free energy change prediction in some selected non-synonymous SNPs (nsSNP) using I-Mutant in PTX-3 protein**.

**nsSNP ID**	**Mutation position**	**DDG**	**Sign of DDG**	**RI**	**PTM**	**ConSurf**
rs34655398	H39Q	−1.97	Dec	8		3e
rs143387231	P40S	−1.41	Dec	9		5e
rs148384694	T41I	−0.56	Dec	7		5e
rs3816527	A48D	0.50	Dec	1		7e
rs367899909	V80A	−1.68	Dec	6		2b
rs557539937	A148T	−1.32	Dec	8		1b
rs572907291	A151T	−1.12	Dec	8		3e
rs112277608	T159M	0.43	Dec	0	Yes	3b
rs370211025	L184S	−2.21	Dec	9		8b
rs564774580	R188C	−1.15	Dec	4		9e(F)
rs529759691	K190E	0.64	Dec	3		6e
rs529759691	F193S	−2.58	Dec	8		8e
rs532972316	E235K	−0.87	Dec	6		9e
rS190837481	H269Y	0.51	Dec	1		9b(S)
rs56265729	V246M	−1.03	Dec	8		7b
rs144979346	G306E	0.14	Inc	2		9b(S)
rs4478039	E313K	−0.15	Dec	2		9e(F)
rs76994524	N337S	−0.81	Dec	8		9b(S)
rs146705881	L343I	−0.93	Dec	9		9b
rs140073706	S344R	−0.99	Dec	2		7e
rs373203093	I348V	−1.48	Dec	8		8b
rs138818541	R360W	−0.34	Dec	5		9e(F)

*It shows DDG-change in free energy between mutant and wild; RI, reliability; PTM, Post-translation modification; ConSurf, ConSurf web server used to predict conservation (1–4 variable, 5-Avg, 6–9 conserved) e, exposed; b, indicates buried, and F, S indicates functional or structural residue according to the neural-network algorithm*.

### Prediction of putative phosphorylation, ubiquitylation, and sumoylation sites in PTX-3 protein

Post-translation modifications were investigated in PTX-3 protein as these modifications control various biological processes in cell such as signaling in innate immune pathway and protein–protein interactions (Perkins, 2006; Liu et al., 2013). We used various *in silico* tools to predictive putative post-translation modification sites in PTX-3 protein. To identified amino acids in PTX-3 protein that may undergo phosphorylation, we use NetPhos 2.0 and GPS 2.1 servers. NetPhos 2.0 server predicted 5-serine and threonine- specific sites in PTX-3 protein. No tyrosine site was predicted by this server in PTX-3 protein. Whereas, GPS 2.1 servers predicted 22-serine and 20-threonine specific sites in PTX-3 protein (Table 8). Many of these phosphorylated amino acid residues undergo mutation (Figure 1) and are highly conserved in PTX-3. Some of them are predicted to be functional and structural residue by ConSurf.

**Table 8:** **Putative phosphorylation sites in PTX-3 protein**.

**NetPhos.2**			**GPS 2.1**		
**Serine**	**Threonine**	**Tyrosine**	**Serine**	**Threonine**	**Tyrosine**
28(6b)	44(4e)	Not detected	13(4b)	41(5e)^*^	Not detected
45(5e)	89(4e)		20(2e)	45(5e)	
171(3e)	159(3b)^*^		54(7e)	77(1b)	
260(5e)	223(6b)		66(9b)S^*^	114(1e)	
274(9e)F	281(2b)		98(1e)	124(4e)	
			115(1e)	159(3b)^*^	
			173(1e)	181(8b)	
			*189(9b)S*	216(9b)S^*^	
			**195(8e)F**	*222(9b)S*	
			206(7b)	**229(9e)F**	
			208(9b)	268(3b)	
			*226(9b)S*	273(5e)	
			241(6b)^*^	281(2b)	
			244(7b)	292(4b)	
			263(2b)	293(2b)	
			**276(9e)F**	298(1b)	
			282(6b)^*^	326(4b)	
			**330(8e)F**	**334(9e)F**	
			341(4e)	351(4e)	
			344(7e)^*^	*369(9b)S*	
			356(7e)		
			380(5e)		

It represents the different amino acids with phosphorylation sites. Conservation scores were calculated by ConSurf and given under brackets along with exposed and buried indication. Functional putative residues indicated as bold and structural putative residues indicated as italicized. nsSNPs indicated as asterisk (^*^).

In addition to phosphorylation site, we also identified ubiquitylation in PTX-3 protein by use of UbPred and BDM-PUB servers. Only single amino acid residue 314 was found, which undergo ubiquitylation (Table 9). We also screened the PTX-3 protein for sumoylation sites using SUMOsp 2.0 and SUMOplot but none of the amino acid was found, which undergo sumoylation but only amino acids that undergo sumo interaction were identified by SUMOsp 2.0 (Table 9).

**Table 9:** **Putative sumoylation and ubiquitylation sites in PTX-3 protein**.

**Sumoylation**		**Ubiquitylation**	
**SUMOplot**	**SUMOsp 2.0**	**BDM-PUB**	**UbPred**
58(9e)F	**237(9e)F**	190(6e)	58(9e)F
191(8e)F	**238(9b)S**	191(8e)F	190(6e)
314(8e)F	**239(7b)**	221(8e)F	191(8e)F
	240(8b)^*^	230(7e)	214(9e)F
	241(6e)^*^	255(3e)	221(8e)F
			230(7e)
			255(3e)
			**314(8e)F**

It indicates the amino acids with sumoylation and ubiquitylation. No sumoylation of any amino acid residue detected by SUMOplot. Whereas in case of SUMOsp 2.0 only amino acids undergo sumo interaction were detected and indicated in bold. Only one putative ubiquitylation site was detected by two programs, indicated in bold. Conservation score for amino acids given in bracts, where “F” indicates functional and “S” indicates structural residue according to the neural-network algorithm. nsSNPs indicated as asterisk (^*^).

Previously, it has been observed that glycosylation of 220 amino acid residues of PTX-3 protein leads to increase in molecular weight of this protein from 40 kDa to 45 kDa. Unique glycosylation sites patterns are associated with different inflammatory cell and induce PTX-3 production by different innate immune cell (Balhara et al., 2013). Thus, post-translation modification sites are important for PTX-3 protein to regulate innate immune response.

### Protein-protein interactions study of PTX-3 protein

**Figure 5:**
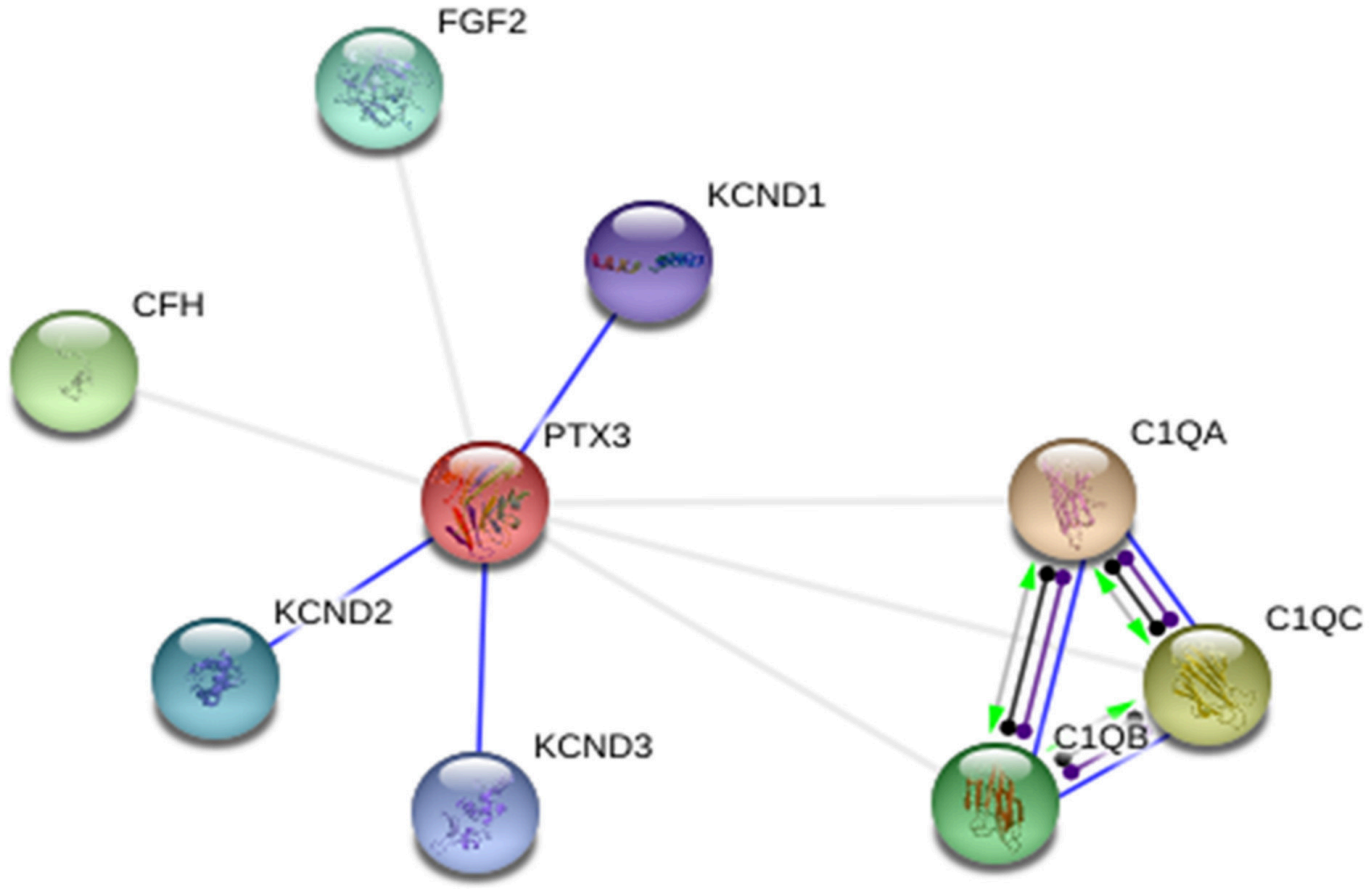
**PTX-3 protein–protein interactions in action view**. It showed predicted binding interaction of PTX-3 with KCND1 (potassium voltage-gated channel, Shal-related subfamily, member (1), KCND2 (potassium voltage-gated channel, Shal-related subfamily, member (2), and KCND3 (potassium voltage-gated channel, Shal-related subfamily, member (3). Association of PTX-3 was observed with C1qA (complement component 1, q subcomponent, A chain), C1qB (complement component 1, q subcomponent, B chain), C1qC (complement component 1, q subcomponent, C chain), CFH (complement factor H), and FGF2 (fibroblast growth factor 2).

STRING database was used to annotate PTX-3 protein interaction with other proteins. In interactive mode of STRING database prediction, the binding interaction of PTX-3 protein was observed with only KCND1, KCND2, and KCND3 (Potassium voltage-gated channel, Shal-related subfamily, and members). Whereas, in action view showed association of PTX-3 protein with following proteins; C1qA (complement component 1, q subcomponent, A chain), C1qB (complement component 1, q subcomponent, B chain), C1qC (complement component 1, q subcomponent, C chain), CFH (complement factor H) and FGF2 (Fibroblast growth factor 2) (Figure 5). Further, Nauta et al showed PTX-3 protein interacts with complement pathway components C1q and activates the classical complement pathway (Nauta et al., 2003). If high risk deleterious nsSNP is present in pentraxin-domain, interaction of PTX-3 protein with C1q component could be affected.

## Conclusion

We conclude that several nsSNPs are present in PTX-3 gene. Most of these nsSNPs are located into pentraxin-domain of PTX-3 protein. Structural analysis of selected high-risk nsSNPs showed that the amino acid residue substitutions in pentraxin-domain had the deleterious impact on the stability of the PTX-3 protein. Amino acid residues which undergo substitution were E313K (rs4478039), N337S (rs76994524), R188C (rs564774580) and R360W (rs138818541). Overall, these nsSNPs showed deleterious effect on structure and subsequently to the function of PTX-3 protein. As, pentraxin-domain of pentraxin protein family previously known to interact with components of complement pathway and activates them and also function as pathogen recognition receptor for these pathogens e.g., *A. fumigatus* conidia, *P. aeruginosa, S. pneumonia*, and *S. typhimurium*. Due to the presence of these high-risk nsSNPs, it could be possible that the domain may not be functionally active and humans with these nsSNPs in their genome may be susceptible to infection for selected pathogens. Finally, we propose that these nsSNPs should be considered for risk assessment against infectious microbes in a population based study.

## Author contributions

Conceived and designed the experiments: RT and JS. Performed the experiments: RT. Analyzed the data: RT and JS. Contributed reagents/materials/analysis tools: JS. Wrote the paper: RT and JS.

### Conflict of interest statement

The authors declare that the research was conducted in the absence of any commercial or financial relationships that could be construed as a potential conflict of interest.

## Supplementary material

Supplementary Figure 1 **Consurf results showing conservation and functional profile for PTX-3 protein**.

Supplementary Figure 2 **Rampage result of wild type PTX-3 protein structure**.

Supplementary Figure 3 **Rampage result of mutated R188C PTX-3 protein structure**.

Supplementary Figure 4 **Rampage result of E313K PTX-3 protein structure**.

Supplementary Figure 5 **Rampage result of N337S PTX-3 protein structure**.

Supplementary Figure 6 **Rampage result of R360W PTX-3 protein structure**.

Supplementary Table 1 **Prediction of deleterious nsSNP in PTX-3 using seven different algorithms**.
