# Supplementary material for: In silico Analysis Revealed High-risk Single Nucleotide Polymorphisms in Human Pentraxin-3 Gene and their Impact on Innate Immune Response against Microbial Pathogens
Source: Front Microbiol. 2016 Feb 23;7:192. doi: 10.3389/fmicb.2016.00192 (PMC4763014; doi:10.3389/fmicb.2016.00192)
Supplement: Supplementary Figure 1 — Consurf results showing conservation and functional profile for PTX-3 protein. [file Image1.PDF]

ConSurf Results

```
1            11           21           31           41
MHLLAILFCA   LWSAVLAENS   DDYDLMYVNL   DNEIDNGLHP   TEDPTPCACG
eebbbbbbbb   bbbbbeeeee   eebebbbbbb   eeebeeeeee   eeeeeebebe
f                                       ff

51           61           71           81           91
QEHSEWDKLF   IMLENSQMRE   RMLLQATDDV   LRGELQRLRE   ELGRLAESLA
eeeeebeebb   bbbeebebee   ebbbebbeeb   beeebeebee   ebeebbeebe
     sff s    s f sfsff       f

101          111          121          131          141
RPCAPGAPAE   ARLTSALDEL   LQATRDAGRR   LARMEGAEAQ   RPEEAGRALA
eebeeeeeee   eeeeebbeeb   beeeeeeeee   beebeeeeee   eeeeeeebbe

151          161          171          181          191
AVLEELRQTR   ADLHAVQGWA   ARSWLPAGCE   TAILFPMRSK   KIFGSVHPVR
ebbeebeebe   eebeebeeeb   eeeebeeebe   bbbbbebebe   ebbbebbbee
                                   f    s  sfsfs    f   fs s

201          211          221          231          241
PMRLESFSAC   IWVKATDVLN   KTILFSYGTK   RNPYEIQLYL   SYQSIVFVVG
ebebebbbbb   bbbebbebbe   ebbbbbebee   eeeeebebbb   eeebbbbbbb
       s s    s f s   f   fs  ssfsf     ff f fs             s

251          261          271          281          291
GEENKLVAEA   MVSLGRWTHL   CGTWNSEEGL   TSLWVNGELA   ATTVEMATGH
eeeeebebee   bbbebebbbb   bbeeeeeeee   bbbbbebeeb   bbbbebbeeb
                   s s    s  f f  f      ss

301          311          321          331          341
IVPEGGILQI   GQEKNGCCVG   GGFDETLAFS   GRLTGFNIWD   SVLSNEEIRE
bbeeebbbeb   eeeeeeebee   eeeeebbebe   bebebbbbbb   ebbeeeebee
  f fs  f    fffff          fff  fsf   sf f  s s      s

351          361          371          381
TGGAESCHIR   GNIVGWGVTE   IQPHGGAQYV   S
eeeeeebebe   bebbbbbbbe   beeeeeeeee   e
      s  f   sf s ss sf    f  fffff    f
```

The conservation scale:

1	2	3	4	5	6	7	8	9

Variable Average Conserved

e - An exposed residue according to the neural-network algorithm.
b - A buried residue according to the neural-network algorithm.
f - A predicted functional residue (highly conserved and exposed).
s - A predicted structural residue (highly conserved and buried).
