# Supplementary material for: In silico Analysis Revealed High-risk Single Nucleotide Polymorphisms in Human Pentraxin-3 Gene and their Impact on Innate Immune Response against Microbial Pathogens
Source: Front Microbiol. 2016 Feb 23;7:192. doi: 10.3389/fmicb.2016.00192 (PMC4763014; doi:10.3389/fmicb.2016.00192)
Supplement: Supplementary Figure 4 — Rampage result of E313K PTX-3 protein structure. [file Image4.PDF]

E313K

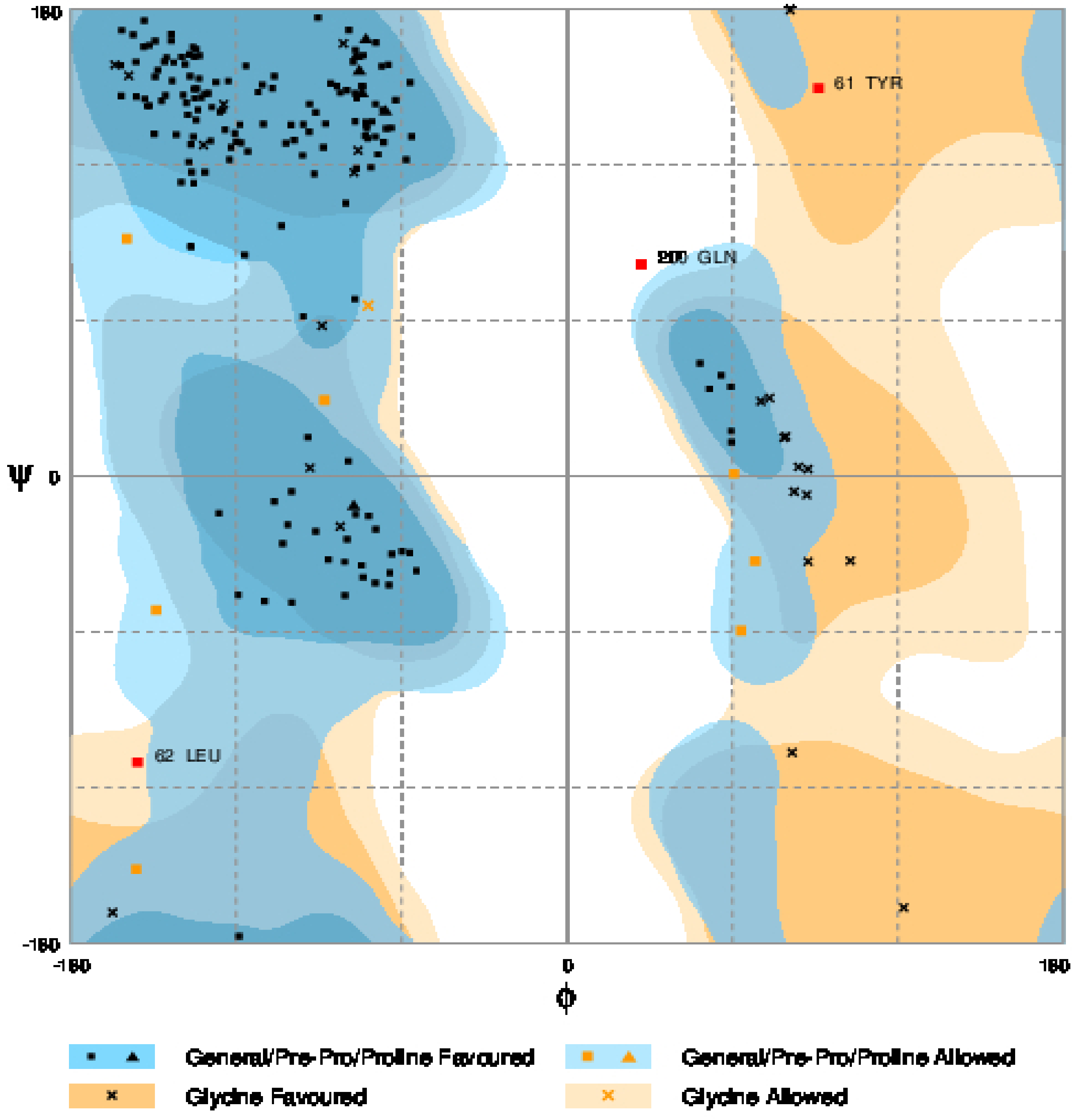

180
-180
0
180
ψ
φ
61 TYR
200 GLN
62 LEU
General/Pre-Pro/Proline Favoured
General/Pre-Pro/Proline Allowed
Glycine Favoured
Glycine Allowed
