# Supplementary material for: In silico Analysis Revealed High-risk Single Nucleotide Polymorphisms in Human Pentraxin-3 Gene and their Impact on Innate Immune Response against Microbial Pathogens
Source: Front Microbiol. 2016 Feb 23;7:192. doi: 10.3389/fmicb.2016.00192 (PMC4763014; doi:10.3389/fmicb.2016.00192)
Supplement: Supplementary Figure 5 — Rampage result of N337S PTX-3 protein structure. [file Image5.PDF]

N337S

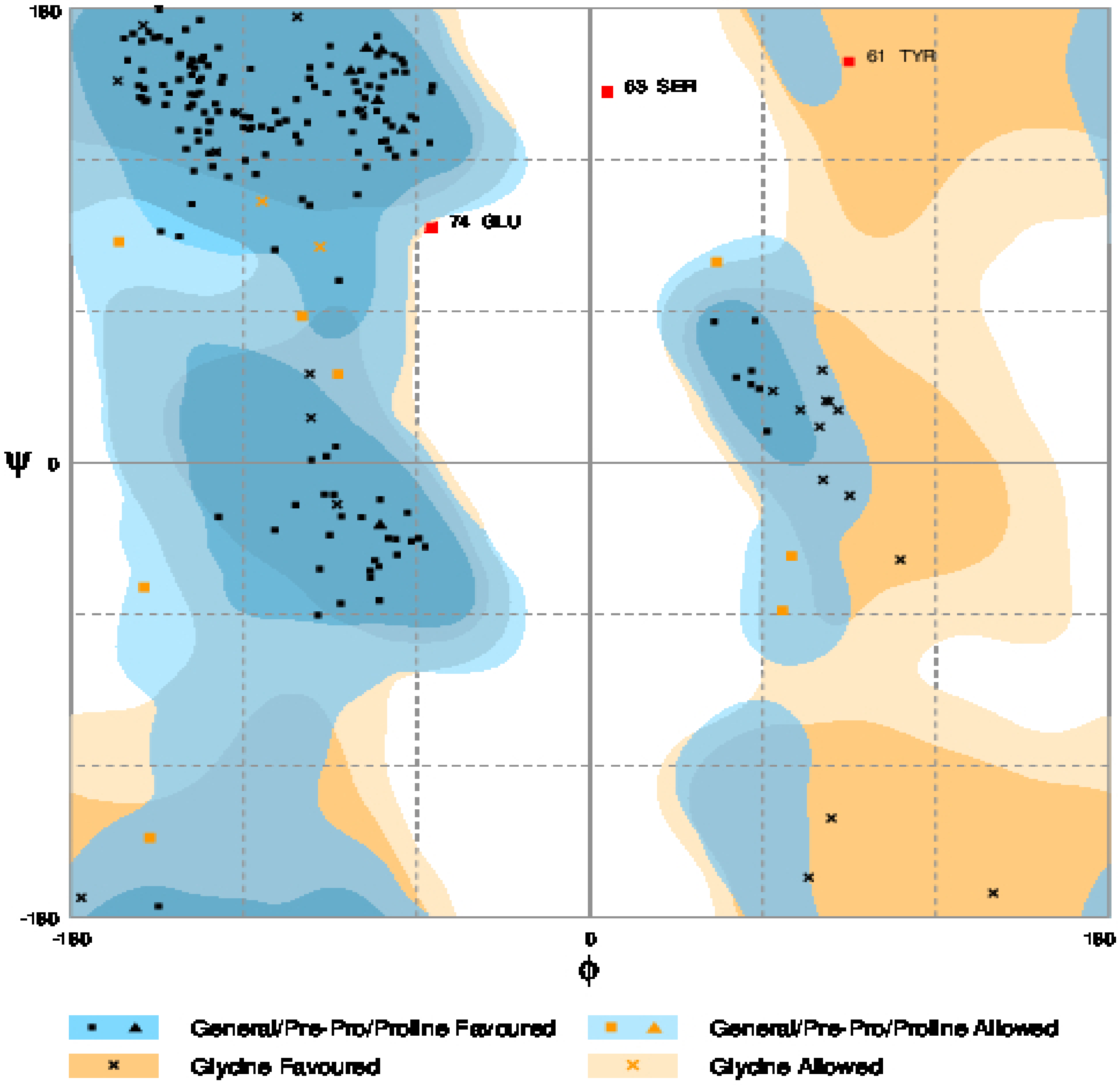

180
Ψ 0
-180
-180
0
180
Φ
61 TYR
63 SER
74 GLU
General/Pre-Pro/Proline Favoured
General/Pre-Pro/Proline Allowed
Glycine Favoured
Glycine Allowed
