# Supplementary figures and images for: In silico Analysis Revealed High-risk Single Nucleotide Polymorphisms in Human Pentraxin-3 Gene and their Impact on Innate Immune Response against Microbial Pathogens
Source: Front Microbiol. 2016 Feb 23;7:192. doi: 10.3389/fmicb.2016.00192 (PMC4763014; doi:10.3389/fmicb.2016.00192)

R360W

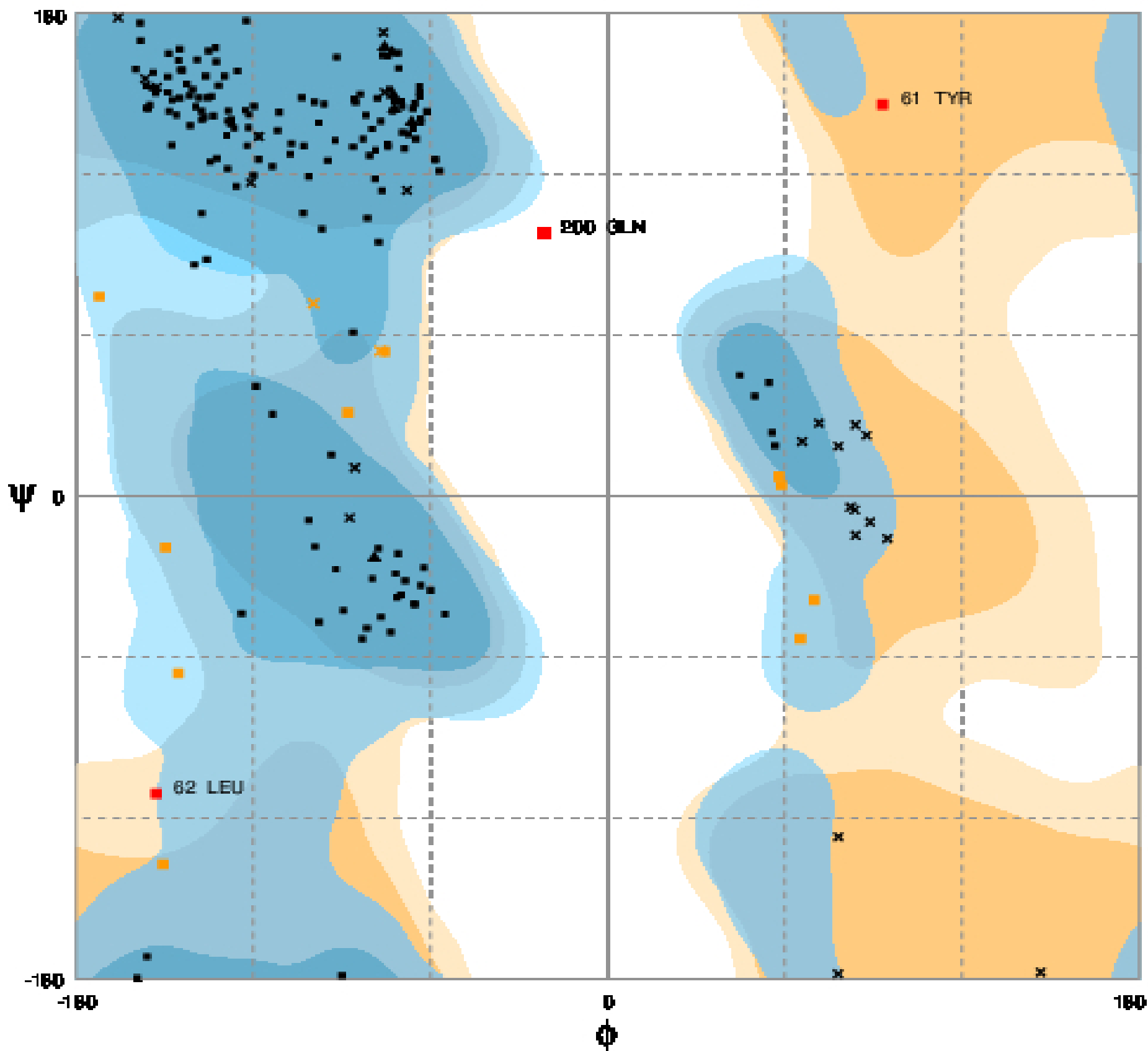

Supplement: Supplementary Figure 6 — Rampage result of R360W PTX-3 protein structure. [file Image6.PDF]
