# Supplementary material for: In silico Analysis Revealed High-risk Single Nucleotide Polymorphisms in Human Pentraxin-3 Gene and their Impact on Innate Immune Response against Microbial Pathogens
Source: Front Microbiol. 2016 Feb 23;7:192. doi: 10.3389/fmicb.2016.00192 (PMC4763014; doi:10.3389/fmicb.2016.00192)
Supplement: Supplementary Table 1 — Prediction of deleterious nsSNP in PTX-3 using seven different algorithms. [file Table1.PDF]

Supplementary table-1

Prediction of deleterious nsSNP in PTX-3

This table represents the number of deleterious nsSNPs predicted by different combination of SNP prediction algorithms. If we use more stringent criteria i.e., 7 out of 7 in silico SNP prediction algorithms very less number of deleterious nsSNPs were predicted.

Number of SNP prediction algorithms	Number of deleterious prediction
3 SNP prediction algorithms	13
4 SNP prediction algorithms	11
5 SNP prediction algorithms	10
6 SNP prediction algorithms	7
7 SNP prediction algorithms	2

3 SNP prediction algorithms (PP-1, PP-2, PhD-SNP), 4 SNP prediction algorithms (PP-1, PP-2, PhD-SNP, SIFT), 5 SNP prediction algorithms (PP-1, PP-2, PhD-SNP, SIFT, SNAP), 6 SNP prediction algorithms (PP-1, PP-2, PhD-SNP, SIFT, SNAP, MAPP), 7 SNP prediction algorithms (PP-1, PP-2, PhD-SNP, SIFT, SNAP, MAPP, PANTHER)
